# The Impact of Non-Pharmacological Interventions on Delirium in Neurological Intensive Care Unit Patients: A Single-Center Interrupted Time Series Trial

**DOI:** 10.3390/jcm12185820

**Published:** 2023-09-07

**Authors:** Paul J. T. Rood, Dharmanand Ramnarain, Annemarie W. Oldenbeuving, Brenda L. den Oudsten, Sjaak Pouwels, Lex M. van Loon, Steven Teerenstra, Peter Pickkers, Jolanda de Vries, Mark van den Boogaard

**Affiliations:** 1 Department of Intensive Care Medicine, Radboud University Medical Center, P.O. Box 9101, 6500 HB Nijmegen, The Netherlands; 2 Research Department of Emergency and Critical Care, School of Health Studies, HAN University of Applied Sciences, P.O. Box 6960, 6503 GL Nijmegen, The Netherlands; 3 Department of Intensive Care Medicine, Elisabeth Tweesteden Hospital, Hilvarenbeekseweg, P.O. Box 90151, 5000 LE Tilburg, The Netherlands; 4 Department of Medical and Clinical Psychology, Center of Research on Psychological Disorders and Somatic Diseases (CoRPS), Tilburg University, P.O. Box 90153, 5000 LE Tilburg, The Netherlands; 5 Department of General and Abdominal Surgery, Helios Klinikum, Lutherplatz 40, 47805 Krefeld, Germany; 6 College of Health and Medicine, Australian National University, 131 Garran Rd, Acton, Canberra, ACT 2601, Australia; 7 Department for Health Evidence, Section Biostatistics, Radboud University Medical Center, P.O. Box 9101, 6500 HB Nijmegen, The Netherlands; 8 Radboud Center for Infectious Diseases, Radboud Institute for Molecular Life Sciences, Radboud University Medical Center, P.O. Box 9101, 6500 HB Nijmegen, The Netherlands; 9 Admiraal de Ruyter Hospital (Adrz), P.O. Box 15, 4462 RA Goes, The Netherlands

**Keywords:** delirium, nursing, critical care, ICU, neurology, non-pharmacologic interventions

## Abstract

**Background**: Delirium is a pathobiological brain process that is frequently observed in Intensive Care Unit (ICU) patients, and is associated with longer hospitalization as well as long-term cognitive impairment. In neurological ICU patients, delirium may be more treatment-resistant due to the initial brain injury. This study examined the effects of a multicomponent non-pharmacological nursing intervention program on delirium in neurological ICU patients. **Methods**: A single-center interrupted time series trial was conducted in adult neurological ICU patients at high risk for developing delirium who were non-delirious at admission. A multicomponent nursing intervention program focusing on modifiable risk factors for delirium, including the optimalization of vision, hearing, orientation and cognition, sleep and mobilization, was implemented as the standard of care, and its effects were studied. The primary outcome was the number of delirium-free and coma-free days alive at 28 days after ICU admission. The secondary outcomes included delirium incidence and duration, ICU and hospital length-of-stay and duration of mechanical ventilation. **Results**: Of 289 eligible patients admitted to the ICU, 130 patients were included, with a mean age of 68 ± 11 years, a mean APACHE-IV score of 79 ± 25 and a median predicted delirium risk (E-PRE-DELIRIC) score of 42 [IQR 38–50]). Of these, 73 were included in the intervention period and 57 in the control period. The median delirium- and coma-free days alive were 15 days [IQR 0–26] in the intervention group and 10 days [IQR 0–24] in the control group (level change −0.48 days, 95% confidence interval (95%CI) −7 to 6 days, *p* = 0.87; slope change −0.95 days, 95%CI −2.41 to 0.52 days, *p* = 0.18). **Conclusions**: In neurological ICU patients, our multicomponent non-pharmacological nursing intervention program did not change the number of delirium-free and coma-free days alive after 28 days.

## 1. Introduction

Delirium is a pathobiological brain process that is frequently observed in Intensive Care Unit (ICU) patients [1]. In patients with traumatic brain injury, high delirium prevalence of up to 88% has been reported [2]. The pathophysiology of delirium is multi-factorial [3], while in neurological patients, apart from neuroinflammatory and neurotransmitter imbalances, there may be additional anatomical and functional brain injuries that may contribute to delirium [4].

Currently, there are a limited number of studies exploring delirium in neurological patients. Delirium in these patients is often unrecognized and interpreted as agitation or a low level of consciousness attributed to the initial brain injury [5,6]. Therefore, in most delirium studies, neurological patients are excluded and adequate treatment options are lacking, especially for neurological patients in the ICU [7]. Antipsychotic medications, such as haloperidol, are ineffective in the prevention and treatment of delirium, and are therefore currently only recommended for ICU patients who experience hallucinations, delusion-associated fearfulness or agitation [7,8].

The use of multicomponent non-pharmacological interventions to improve outcomes in general ICU patients with delirium has been suggested [7,9,10,11]. Some of these interventions, for instance, the optimization of audiovisual function, cognitive functioning, sleep and mobilization, are commonly applied by attending ICU nurses. However, the effectiveness of a multicomponent program has not been studied in neurological ICU patients.

Recently, the results of a large multicenter stepped-wedge cluster-randomized controlled trial to evaluate the effectiveness of multicomponent non-pharmacologic interventions in non-neurological ICU patients were published [12]. In one of the participating centers, neurological ICU patients were also included.

The aim of this study was to examine the effect of the use of a multicomponent program of non-pharmacological nursing interventions on the number of delirium-free and coma-free days alive in neurological ICU patients.

## 2. Methods

### 2.1. Design

A single-center, interrupted time series (ITS) (where the moment of introducing the intervention was randomized) trial in neurological ICU patients was conducted within a larger stepped-wedge cluster-randomized trial between 31 December 2016 and 1 May 2019 [12]. It was carried out in accordance with the applicable rules concerning the review of research ethics committees and informed consent in the Netherlands (MREC Arnhem-Nijmegen, 2013/173). All patients or legal representatives were informed about the details of this study and could decline to participate. A study protocol was previously published [13]. After the study onset, no methodological changes were made.

### 2.2. Participants

Adult ICU patients admitted for a neurological reason who were expected to stay for at least 24 h and were at high risk of developing delirium (defined as an E-PRE-DELIRIC score [14] of ≥35%), and who were delirium-free at the time of ICU admission, were eligible. Patients were excluded when reliable assessment for delirium was not possible (due to persistent coma, audiovisual disorders, language problems, mental disability, or aphasia).

### 2.3. Interventions and Implementation

The UNDERPIN-ICU program focused on optimizing the following modifiable delirium risk factors: visual and hearing impairment (e.g., use of visual and hearing aids whenever awake, approaching from the best side for vision and hearing), orientation loss (e.g., providing a schedule, clock and calendar for each patient, promotion of the provision of personal objects), sleep deprivation (e.g., optimizing circadian rhythm, noise reduction), cognitive impairment (e.g., provision of cognitive training exercises) and immobility (e.g., encouraging clear mobilization goals, minimizing sedation and physical restraints), which have been proven effective in non-ICU patients [15,16,17], and were customized specifically for ICU patients. Nurses were also encouraged to apply the program for delirium prevention as well as minimizing delirium after onset, and were allowed to individually tailor the program to patients’ needs, while covering all domains as much as possible (Table 1, Supplemental file S1) [12,13].

During the control period, some non-pharmacological delirium-preventive interventions may have already been applied, although unstructured and in the absence of a local delirium prevention protocol. After a control period of 15 months, the intervention program was implemented as the standard of care. After a two-month training period, the program was considered standard care and the intervention period, which consisted of 17 months, started. During the training period, the intervention program was implemented as a local non-pharmacological delirium prevention and treatment protocol, and a coalition group was formed that consisted of a physician, several nurses and supporting staff, who were selected based on their motivation to reduce delirium as well as previous experience with local implementation projects. The preformatted intervention program materials were adjusted to the local standards and nurses and medical staff were trained. The coalition group led the implementation strategy, which focused on education on interventions at the patient, provider and organizational levels, as well as motivational strategies [18]. Plasticized posters and practical intervention materials were hung to enhance awareness. Feedback on noise was realized through a sound level monitoring stoplight (Yacker Tracker, Attention Getters Inc., El Cajon, CA, USA) for two months after the implementation of the program. Also, coffee mugs containing the intervention program pictograms were used during coffee breaks as daily reminders. Regular study information and updates were communicated via posters and newsletters. Three monthly newsletters were distributed that updated readers on the progress of the study, and specific local feedback was given on compliance with the application of cognitive training exercises and light and sound levels for adequate enforcement of the execution of the program (Supplemental file S2).

### 2.4. Outcomes

The primary outcome was the number of delirium-free and coma-free days alive in the 28 days after ICU admission. The secondary outcomes were delirium incidence, delirium duration, 28-day and 90-day mortality, the duration of mechanical ventilation, re-intubations, ICU re-admissions and the accidental removal of tubes/catheters, the use of physical restraints, and length of stay in the ICU and hospital [13].

### 2.5. Data Collection

The screening and administrative compliance, as well as use of the specifically developed UNDERPIN-ICU application for cognitive training, were monitored in real time via tablet computers (iPad Air 2, Apple, Cupertino, CA, USA). A web-based application was developed (PIECA, Beagleboxx, Amsterdam, the Netherlands) that contained weblinks to online questionnaires (SurveyMonkey, San Mateo, CA, USA). The app also provided e-learning, screening and treatment tools, and was used for sharing information about the intervention program, the three-monthly newsletters and recreational features such as television, newspapers, magazines and puzzles.

Delirium was monitored using the Confusion Assessment Method for the Intensive Care Unit (CAM-ICU) [19] including the Richmond Agitation and Sedation Scale (RASS) [20], which was executed at least once during every 8 h shift. On a daily basis, the Numeric Rating Scale for Sleep (NRS Sleep) [21] and Nursing Activities Score (NAS) [22] were used to monitor sleep quality and nursing workload.

To monitor compliance with the domain of minimizing sleep deprivation, decibel and lux levels were monitored in two different rooms per ICU, at 1 Hz (every second) (VitalMinds, Royal Dutch Philips, Eindhoven, The Netherlands) (Supplemental file S2), for which an additional MREC waiver was obtained (MREC Arnhem-Nijmegen, 2017-3193). All systems used complied with local security and privacy standards.

### 2.6. Statistical Analyses

Descriptive statistics and process measures are presented as means (± standard deviation) or medians [25th–75th Inter Quartile Range (IQR)], based on their distribution for continuous variables, or frequencies for categorical variables. After assessment of normality, an independent samples *t*-test or Mann–Whitney U test was used, as appropriate. To perform the interrupted time series analyses, continuous and binary patient-level outcomes in the time periods were first aggregated to the ICU average in those periods, corresponding with the periods defined in the encompassing UNDERPIN-ICU trial. Based on the few missing data (0.2%), no imputation was performed. Outcomes with a skewed distribution (e.g., coma days) were log-transformed before aggregating. For binary outcomes, the period averages were log-odds-transformed, after replacing 0% and 100% with 0.0001% and 99.9999% to avoid infinities in the log-odds transformation. Then, the resulting time series was analyzed using linear autoregression to account for correlation between ICU averages and to estimate the effect between before and after the introduction of the intervention. Ordinary least squares regression (no correction for possible correlation), autoregressive regression for each lag order (estimated using maximum likelihood) and backward autoregression (backward selection based the Yule–Walker estimates) were performed. The fits of these models were compared in terms of residuals plots (no indication of alternation or long sequences of only positive or only negatives residuals), fit measure (corrected Akaike Information Criterion (AIC)), and white noise probabilities. The estimates of the level change and the slope change of the best-fitting interrupted time series model were reported. For binary outcomes, these effects were back-transformed to odds ratios (ORs). For continuous outcomes, the effects describe the difference in averages (level and slope) if the outcome was not log-transformed. For log-transformed outcomes, the effects are given as median ratios after back-transformation. A per-protocol analysis was conducted, which excluded patients who actually did not meet the inclusion criteria, or were included during the training period because of possible contamination effects. For this sub study, no a priori power analysis was performed. All outcome data were analyzed by an independent statistician (ST). Data were analyzed using IBM SPSS Statistics 25, SAS 9.2, Matlab R2019b and GraphPad Prism 8.3. Statistical significance was defined as *p* < 0.05.

## 3. Results

**Figure 1:**
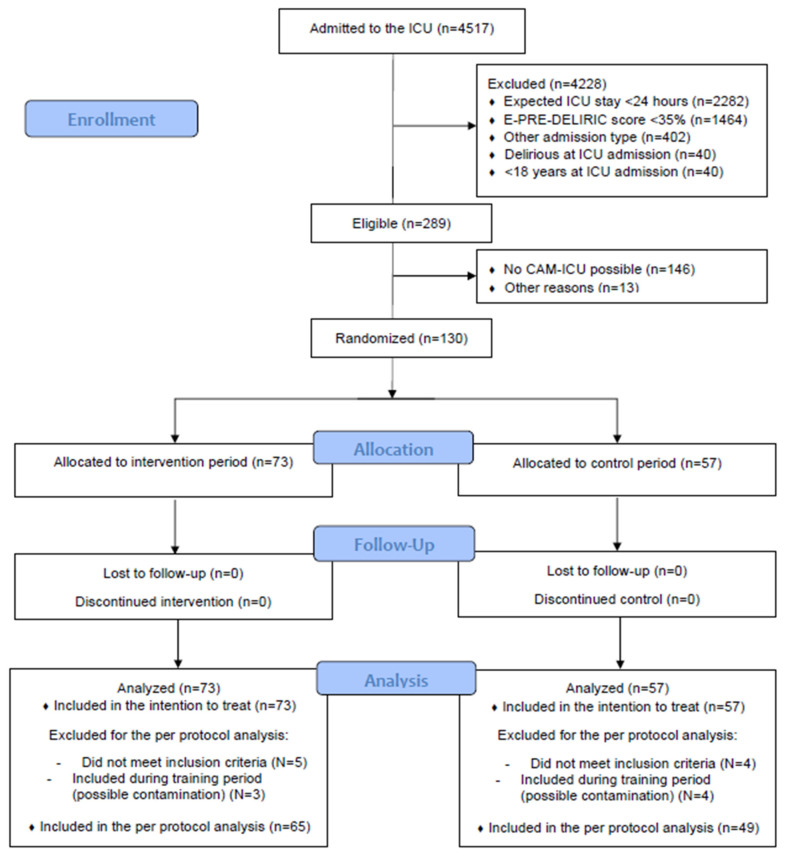
Flow chart.

During the study period, 289 eligible patients were admitted to the ICU, of which 130 patients were included (Figure 1). The main diagnoses were predominantly neuro trauma (46, 35%), intracerebral hemorrhage (22, 17%), acute subarachnoid hemorrhage (21, 16%) and cerebrovascular incidents (13, 10%). The patients had a mean age of 68 (±11) years, 72 patients (55%) were male, and the mean APACHE-IV score [23] was 79 (±25). A total of 73 patients were included in the intervention period, and 57 patients in the control period. Between groups, only the predicted delirium risk (E-PRE-DELERIC score [14]) significantly differed, with a mean of 41 (±11) in the intervention group versus 46 (±10) in the control group (*p* = 0.01). During the study, no adverse effects of the multicomponent intervention program were reported (Table 2).

### 3.1. Primary Outcome

**Figure 2:**
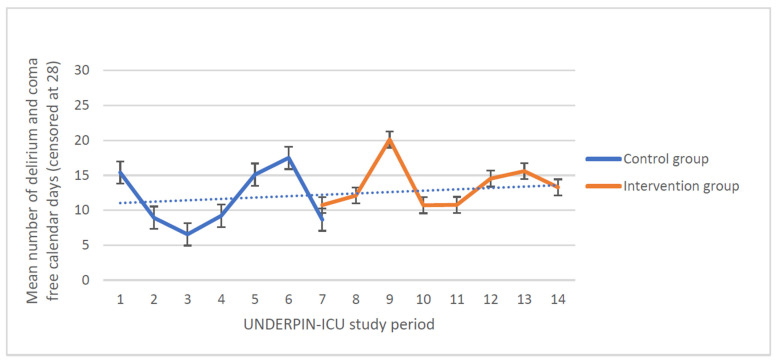
Mean number of delirium-free and coma-free days alive in 28 days (interrupted time series analysis) during the control period and during the intervention period. A study period consisted of two months.

The median number of delirium-free and coma-free days alive in 28 days was 15 [IQR 0–26] days in the intervention period and 10 [0–24] days in the control period (level change −0.48 days, 95% confidence interval (95%CI) −7.07 to 6.10 days, *p* = 0.87; slope change −0.95 days, 95%CI −2.41 to 0.52 days, *p* = 0.18) (Table 3). The interrupted time series analysis of delirium-free and coma-free days alive is shown in Figure 2.

### 3.2. Secondary Outcomes

Similar results were determined in all secondary outcomes: 33 patients (24%) developed delirium in the intervention period, and 74 patients (74%) in the control period (odds ratio (OR) level change 11.38, 95%CI < 0.001 to 26,647, *p* = 0.50; OR slope change 4.67, 95%CI 0.66 to 32.75, *p* = 0.11). In delirium incident cases, the median [IQR] number of delirium days was 2 [1–7] days in the intervention period, and 2 [2–4] days in the control period (median ratio (MR) level change 1.59, 95%CI 0.72 to 3.49, *p* = 0.22; MR slope change 1.21, 95%CI 1.00 to 1.48, *p* = 0.05). The number of coma days was 3 [1–5] days in the intervention period, and 2 [0–4] days in the control period (MR level change 1.29, 95%CI 0.69 to 2.43, *p* = 0.39; MR slope change 1.10, 95%CI 0.94 to 1.29, *p* = 0.21). Mortality at 28 days was not significantly different (33 patients (45%) in the intervention period, and 25 patients (44%)) (OR level change 0.01, 95%CI < 0.001 to 35.79, *p* = 0.24; OR slope change 0.16, 95%CI 0.02 to 1.24, *p* = 0.07) (Table 3).

### 3.3. Process and Compliance Measures

The median RASS levels remained similar during all shifts: −1 [−3–0] in the intervention period, and −1 [−3–0] in the control period (*p* = 0.37). Also, the quality of sleep in patients remained similar: a mean NRS Sleep score [21] of 6 (±2) was reported in the intervention period, and 6 (±2) was reported in the control period (*p* = 0.13).

Light (LUX) levels significantly changed during nighttime: a median of 9 [9–10] LUX was measured during the intervention period, and 15 [14–17] LUX was measured during the control period (*p* < 0.01). During daytime, LUX levels were not statistically significantly different: a median of 86 [23–133] LUX was measured during the intervention period, and 40 [21–109] LUX was measured during the control period (*p* = 0.64). Noise levels (dB) were significantly different: median 40 [39–41] dB was measured in the intervention period and 43 [43–44] during the control period during daytime (*p* < 0.01), and a median of 39 [38–39] dB was measured in the intervention period and a median of 42 [42–43] dB during the control period during nighttime (*p* < 0.01). The workload of nurses was not significantly changed: the median Nursing Activity Score [22] was 50 [44–64] during the intervention period, and 53 [44–62] during the control period (*p* = 0.45) (Table 4).

## 4. Discussion

In this prospective, interrupted time series with randomized timing of intervention, the use of a multicomponent intervention program in neurological ICU patients with a high risk for delirium did not show significant differences in the number of delirium-free and coma-free days in patients who were still alive after 28 days. Also, no other beneficial effects of the intervention program were found in any of the secondary outcomes.

Despite the advice in a recent ICU guideline [7] suggesting the use of non-pharmacological interventions to reduce delirium burden, our program did not improve the delirium outcome in neurological ICU patients. These results were similar to other studies, which also showed no therapeutic effects in single- or multicomponent interventions [12], even when ICU studies were pooled [24], as well as in another similar primary study using an intervention protocol focused on optimizing sedation, sleep, pain reduction and early mobility in patients with acute brain injury [2].

There may be possible explanations for the lack of effect of nursing intervention protocols and differences in delirium incidence regarding the present study. At the onset of the study, some non-pharmacological delirium-preventive measures were already applied, although they were less structured and not as well defined as in the study protocol. Therefore, the difference between the control period and the intervention period may have been less pronounced than anticipated and this may have influenced the results of this study, as studies in other contexts that report no baseline application of non-pharmacological strategies did report significant effects [25,26].

Furthermore, the pathophysiology of delirium in different neurology patients, including the multiple predisposing factors (e.g., frailty, age, dementia, hypertension) [26,27] and precipitating risk factors (e.g., long and kidney organ failure and coma) [3,28] is complex. Therefore, the sole provision of non-pharmacological interventions may be of limited added value in the prevention and treatment of delirium in neurological ICU patients. Possibly, combining non-pharmacological strategies with pharmacological treatments, such as dexmedetomidine, may prove more effective, as suggested in the A-I delirium prevention bundle [29].

Also, some of the interventions applied may have limited clinical relevance. We were able to reduce noise levels by only 3 dB, which may be clinically ineffective and therefore may be unlikely to influence outcomes, especially as the WHO recommendation is <30–35 dB in patient rooms [30]. However, meaningful differences were found in the LUX levels. In the absence of effective noise control, enhanced sleep hygiene and reduced nursing activity, the sole application of light control did not change the outcomes.

The strengths of this study were the prospective design and the relatively substantial inclusion rate for this sub-population. This is one of very few studies focusing on non-pharmacological multicomponent intervention in the prevention of delirium in neurological ICU patients.

Also, some limitations need to be addressed. First, delirium in this study was assessed using the CAM-ICU and not using the gold standard of DSM-V criteria, which may have underestimated the incidence of delirium in this specific population. However, using the CAM-ICU for delirium assessment in ICU patients is in concordance with, and recommended in, the current Society of Critical Care Medicine’s Pain, Agitation, Delirium, Immobility and Sleep guideline for the ICU [7]. Second, the estimated effect in this study seemed small (−0.48 days in level change), while the raw median effect was large (5 days). In this context, it is important to note that the 95%CI of the level change in the ITS, from −7.07 to 6.10 days (Table 3), may still allow an effect of two to five days (two was the effect considered relevant a priori in the main stepped-wedge study [13]). Given that the SD in the current study was approximately 11.5 days, the a priori power of a study of the same size as the current one would be <12%, assuming a pre–post-comparison without accounting for time trends and autocorrelation). For a difference of 5 days, this would be 48%. With accounting for the correlations and trends, it may be even lower. In this sense, the study had no a priori power, which underpins that adequately powered trials remain needed in the critical care context [31], and should account for the correlations in the data (for example, if the difference is indeed as large as 5 days (SD = 11.5), an ITS analysis of an ICU level with 10 pre and 10 post periods, each having 20 subjects with ICC = 0.01 (so 400 patients in total), and a large autocorrelation of r = 0.95 from one period to the next, would have a power between 73% for an autoregression with lag 1), as well as the relatively large range of delirium- and coma-free days alive determined in this study. As a recent network meta-analysis suggests that multicomponent non-pharmacological interventions seem to be the most effective intervention for ICU delirium prevention [26], additional high-quality and large-scale randomized controlled trials seem warranted. Third, a significant proportion of patients could not be evaluated for delirium because of pharmacological or trauma-induced coma, which may limit the generalizability of the results that were determined. Nevertheless, for the subgroup of patients that are awake, the importance of also determining the effects of non-pharmacological treatment for this specific subpopulation of neurological patients is paramount. As we felt the urgency to also establish these effects, this sub-study was conducted. Fourth, in our study, we only included neurological ICU patients who were at high risk for developing delirium. Possibly, other results may be found in patients with a lower risk for delirium, although sensitivity analyses in other ICU subgroups, including with a low delirium risk, did not indicate beneficial effects in a prior larger multicenter trial [12].

## 5. Conclusions

This study in adult neurological ICU patients at high risk of delirium showed that the use of a non-pharmacological multicomponent intervention aiming to optimize vision, hearing, orientation, sleep, cognition and mobility did not statistically significantly increase the number of delirium-free and coma-free days alive in 28 days compared to standard ICU care. The non-significant median difference of five more delirium- and coma-free days alive may be a positive signal for neurological ICU patients, which should be confirmed in larger randomized trials.

## Figures and Tables

**Table 1:** The UNDERPIN-ICU program summary.

Domain	Activities
*1. Visual and hearing impairment*	Use of visual and hearing aids whenever awake Approach from the best side for vision and hearing Provision of material adapted to patients who have a visual impairment Prevention of cornea dehydration during sedation Attention to verbal communication when severely visually impaired Limiting background noise Use of special communication techniques as appropriate
*2. Sleep deprivation*	Optimizing circadian rhythm Noise reduction Minimizing night-time procedures Providing optimal relaxation Restriction of sleep medication Improving staff awareness of sleep importance Striving to minimize sedation use
*3 Cognitive impairment*	Providing a schedule, clock, and calendar for each patient Promotion of provision of personal objects by next of kin Promoting regular visits Optimizing and tailoring communication based on patients’ preferences Frequent reorientation Provision of cognitive training exercises
*4. Immobility*	Encouraging setting and documenting of clear mobilization goals Minimizing sedation use Minimizing and optimally locating restraining lines Optimizing analgesia and establishing proper guidance for minimizing pain and fear during mobilization Frequent provision of physical therapy and/or mobilizationInvolving next of kin in stimulating early mobilization
*A detailed description of activities can be found in Supplemental file S1*	

**Table 2:** Demographic patient characteristics.

	Intervention N = 73	Control N = 53	*p*-Value
Male/female	36/37 (49/51)	36/21 (63/37)	0.12
Age in years, mean (SD)	69 (11)	67 (11)	0.49
Urgent admission	70 (96)	55 (97)	0.11
APACHE IV score per point, mean (SD)	80 (25)	78 (24)	0.61
History of cognitive disorders	11 (15)	11 (19)	0.60
History of alcohol abuse	15 (21)	11 (19)	0.77
Mean arterial pressure at admission (mmHg), mean (SD)	91 (20)	92 (20)	0.68
Corticosteroids at admission	4 (6)	6 (11)	0.32
Respiratory insufficiency	65 (89)	53 (93)	0.98
Serum urea level at admission (mmol/L), median [IQR]	7 [5 to 9]	5 [5 to 7]	0.10
E-PRE-DELIRIC score, mean (SD)	41 (11)	46 (10)	0.01
RASS score at admission, median [IQR]	−4 [−4 to −3]	−4 [−5 to −2]	0.36
Pre-admission use of visual or hearing aids	18 (25)	13 (23)	0.34

Data are presented as N (%), unless specified otherwise. Abbreviations: SD = standard deviation, APACHE IV = Acute Physiology And Chronic Health Evaluation IV score, IQR = interquartile range, E-PRE-DELIRIC = early prediction model for delirium in ICU patients, RASS = Richmond Agitation and Sedation Scale.

**Table 3:** Outcomes.

	Intervention N = 73	Control N = 57	Level Change (95%CI)	*p*-Value	Slope Change (95%CI)	*p*-Value
Delirium- and coma-free days alive	15 [0–26]	10 [0–24]	−0.48 (−7.07 to 6.10)	0.87	−0.95 (−2.41 to 0.52)	0.18
Delirium days	0 [0–2]	2 [0–4]	1.02 (0.51 to 2.03) *^A^*	0.96	1.11 (0.94 to 1.33) *^A^*	0.19
in delirious patients	2 [1–7]	2 [2–4]	1.59 (0.72 to 3.49) *^A^*	0.22	1.21 (1.00 to 1.48)	0.05
Coma days	3 [1–5]	2 [0–4]	1.29 (0.69 to 2.43) *^A^*	0.39	1.10 (0.94 to 1.29) *^A^*	0.21
Sedation days	2 [1–4]	2 [1–4]	0.95 (0.47 to 1.91) *^A^*	0.87	1.11 (0.93 to 1.33) *^A^*	0.20
Delirium medication days	0 [0–1]	0 [0–3]	3.66 (0.75 to 17.78) *^A^*	0.09	1.36 (0.81 to 2.31) *^A^*	0.19
Delirium incidence	24 (33)	42 (74)	11.38 (<0.001 to 26647) *^B^*	0.50	4.67 (0.66 to 32.75) *^B^*	0.11
Duration of mechanical ventilation	5 [2–11]	4 [2–13]	1.46 (0.87 to 2.45) *^A^*	0.13	1.06 (0.94 to 1.20) *^A^*	0.28
Incidence of re-intubation	15 (21)	9 (16)	0.36 (<0.001 to 4727) *^B^*	0.81	3.52 (0.48 to 26.03) *^B^*	0.18
Incidence of re-admission	4 (6)	8 (14)	0.00 (0.00 to 0.00) *^B^*	<0.001	0.55 (0.23 to 1.30) *^B^*	0.14
Incidence of unplanned removal of tubes/catheters	8 (14)	5 (9)	114.00 (<0.001 to >1000) *^B^*	0.53	7.04 (0.11 to 431.40) *^B^*	0.32
Incidence of physical restraints	33 (45)	37 (65)	1.53 (0.21 to 11.39) *^B^*	0.65	1.07 (0.65 to 1.77) *^B^*	0.77
Duration of physical restraints	0 [0–4]	3 [0–8]	1.18 (0.44 to 3.21) *^A^*	0.71	1.08 (0.84 to 1.39) *^A^*	0.50
ICU length of stay	8 [4–13]	8 [5–18]	1.75 (0.78 to 3.93) *^A^*	0.16	1.77 (0.96 to 1.44) *^A^*	0.10
Hospital length of stay	13 [6–22]	18 [6–34]	1.15 (0.45 to 2.96) *^A^*	0.75	1.08 (0.85 to 1.36) *^A^*	0.51
Mortality, 28 days	33 (45)	25 (44)	0.01 (<0.001 to 35.79) *^B^*	0.24	0.16 (0.02 to 1.24) *^B^*	0.07
Mortality, 90 days	39 (53)	31 (54)	0.006 (<0.001 to 18.28) *^B^*	0.19	0.12 (0.02 to 0.89) *^B^*	0.04

Data are presented as median [interquartile range] or N (%), unless specified otherwise. *^A^* The ratio of medians in the intervention condition to the median in the control condition as estimated from the interrupted time series model after log transformation (see Methods). *^B^* Odds ratio as estimated from the interrupted time series model after log-odds transformation (see Methods). Abbreviation: ICU = Intensive Care Unit.

**Table 4:** Process and compliance data.

		Intervention	Control	*p*-Value
RASS score ^1^	Night	−1 [−3–0]	−1 [−3–0]	0.37
	Day	−1 [−3–0]	−1 [−3–0]	0.86
	Evening	−1 [−3–0]	−1 [−3–0]	0.27
NRS Sleep ^1^		6 (2)	6 (2)	0.13
Light levels (LUX) ^2^	Day	86 [23–133]	40 [21–109]	0.64
	Night	9 [9–10]	15 [14–17]	<0.01
Noise levels (dB) ^2^	Day	40 [39–41]	43 [43–44]	<0.01
	Night	39 [38–39]	42 [42–43]	<0.01
Nursing Activity Score—workload ^1^		50 [44–64]	53 [44–62]	0.45

Data are presented as median [interquartile range] or mean (SD). ^1^ N = 347 daily nursing scores; ^2^ N = 2601 measurement days (1 Hz). Abbreviations: RASS = Richmond Agitation and Sedation Scale, NRS Sleep = Numeric Rating Scale for Sleep Quality.

## Data Availability

The data presented in this study are available on request from the corresponding author.

## Supplementary Materials

The following supporting information can be downloaded at: https://www.mdpi.com/article/10.3390/jcm12185820/s1, Supplemental file S1: Detailed description of the UNDERPIN-ICU program. Supplemental file S2: Light and sound measurements.
