# Supplementary material for: The Impact of Non-Pharmacological Interventions on Delirium in Neurological Intensive Care Unit Patients: A Single-Center Interrupted Time Series Trial
Source: J Clin Med. 2023 Sep 7;12(18):5820. doi: 10.3390/jcm12185820 (PMC10532134; doi:10.3390/jcm12185820)
Supplement: Supplementary file 1 [file jcm-12-05820-s001.zip › jcm-2548824-supplementary.pdf]

Supplemental files

The impact of non-pharmacological interventions on delirium in neurological ICU patients: A single center interrupted time series trial

Supplemental file S1: Detailed description of the UNDERPIN-ICU program

Supplemental file S2: Light and sound measurements

Supplemental file S1: Detailed description of the UNDERPIN-ICU program

A detailed description of the UNDERPIN-ICU program may be found in the supplemental files of the main publication of the UNDERPIN-ICU study (Rood PJT, Zegers M, Ramnarain D, Koopmans M, Klarenbeek T, Ewalds E, van der Steen MS, Oldenbeuving AW, Kuiper MA, Teerenstra S *et al*: The Impact of Nursing Delirium Preventive Interventions in the ICU: A Multicenter Cluster-randomized Controlled Clinical Trial. *Am J Respir Crit Care Med* 2021, 204(6):682-691, doi: 10.1164/rccm.202101-0082OC)

Direct link: https://thoracic-prod-cdn.literatumonline.com/journals/content/ajrccm/2021/ajrccm.2021.204.issue-6/rccm.202101-0082oc/20210916/suppl/rood_data_supplement.pdf?b92b4ad1b4f274c70877518514abb28b4c5b10d83a46107977ccdd07a5f3c131faa840008a4e7c17687f53a8482151b86ec50eaadaad1e41fb1e7e4c16a9e92220cea82b7ec556b48b84368f2ec37b3b015499b08a9689afbf56c52724bd407856970f430a70aa2b188912b954788cc7e254b148b4039ccdf5d1ce787d9cabc88bf063e98c072b978738e7ddf06712061a199e2b328aaba9b7220a08c31d521c

Supplemental file S2: Light and sound measurements

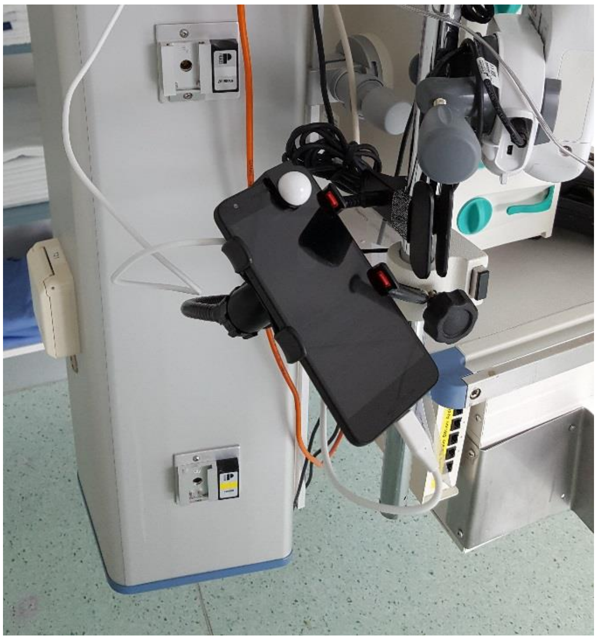

The method for measurement of light and sound levels were published in the main article of the UNDERPIN-ICU study[1]. Briefly, measurements were taken within 1 meter of the patient's head at two separate beds by two smartphones, equipped with a validated commercially available application which sampled decibel and lux levels at 1Hz (VitalMinds, Royal Dutch Philips, the Netherlands). After implementation of the program, the nursing staff regularly received active feedback on sound and lux levels, benchmarked to the other clusters of the main study as well as the World Health Organization international guideline. The mean dB (LaEQ per second) and Lux Levels were stratified for control and intervention period, and plotted over a single 24-hour interval. Differences were tested for 24-hour periods as well as stratified for daytime (06.00-21.59) and nighttime (22.00-05.59) intervals using Mann-Whitney U tests. Data were analyzed using Matlab R2019b (The MathWorks Inc., USA). Graphs were made using GraphPad Prism (GraphPad Software version 8.3, USA).

Results

Measurements were conducted from 2017 to 2019. A total of 224.766.130 samples were taken, reflecting 62435 hours or 2601 measurement days. Median LUX levels did not significantly increase during daytime (median 86 [IQR 23-133] in the intervention period compared to 40 [IQR 21-109] Lux in the control period; p=0.64), but did significantly decrease during nighttime, median 9 [9-10] Lux was observed in the intervention period, compared to 15 [IQR 14-17] in the control period (p<0.01).

[1] Rood PJT, Zegers M, Ramnarain D, et al. The Impact of Nursing Delirium Preventive Interventions in the ICU: A Multicenter Cluster-randomized Controlled Clinical Trial. Am J Respir Crit Care Med 2021;204(6):682-691. (In eng). DOI: 10.1164/rccm.202101-0082OC.

Median noise levels during daytime decreased (median 40 [IQR 39-41] in the intervention period vs. 43 [IQR 43-44] in the control period (p<0.01)), as well as during nighttime (median 39 [38-39] during the intervention period vs. 42 [42-42] in the control period (p=<0.01)).

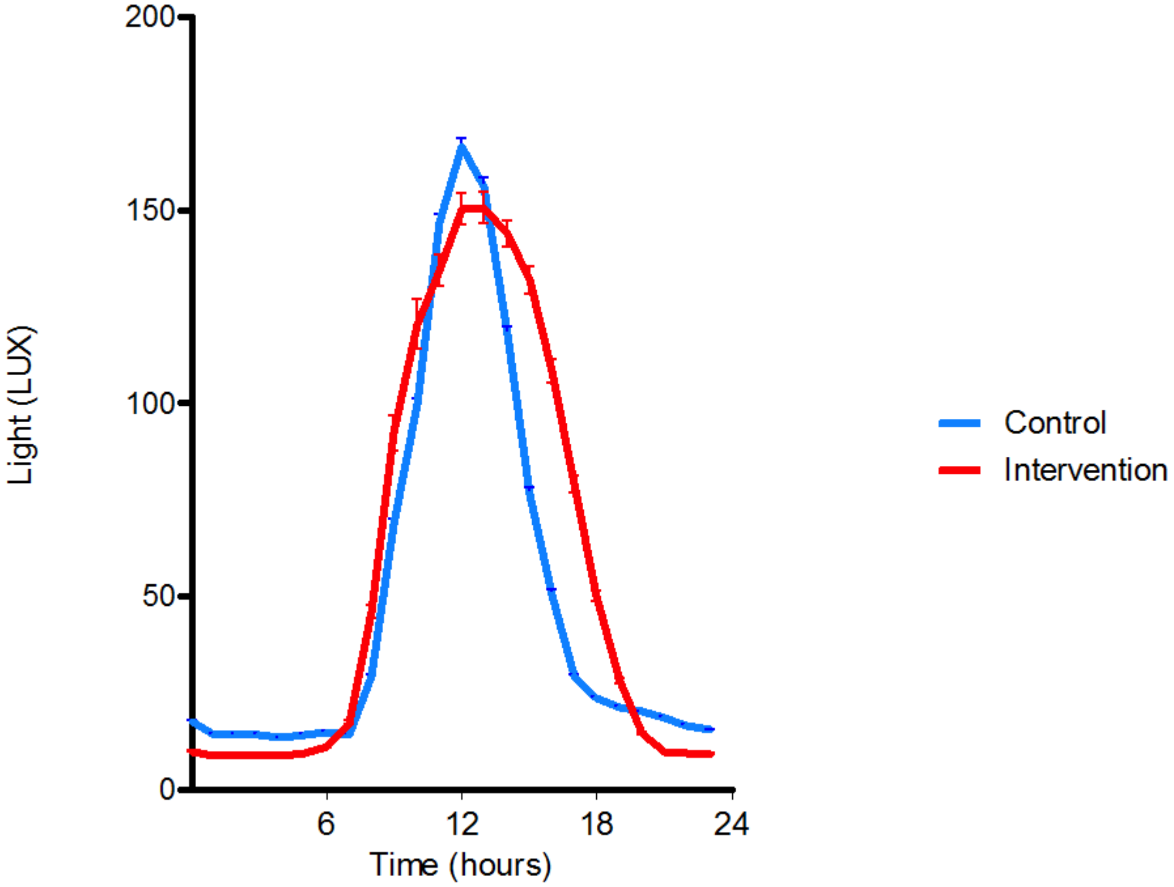

Daily Light levels (LUX) depicted over 24 hours (2 devices, N= 2601 measurement days). Data in the figure are expressed as mean and SEM.

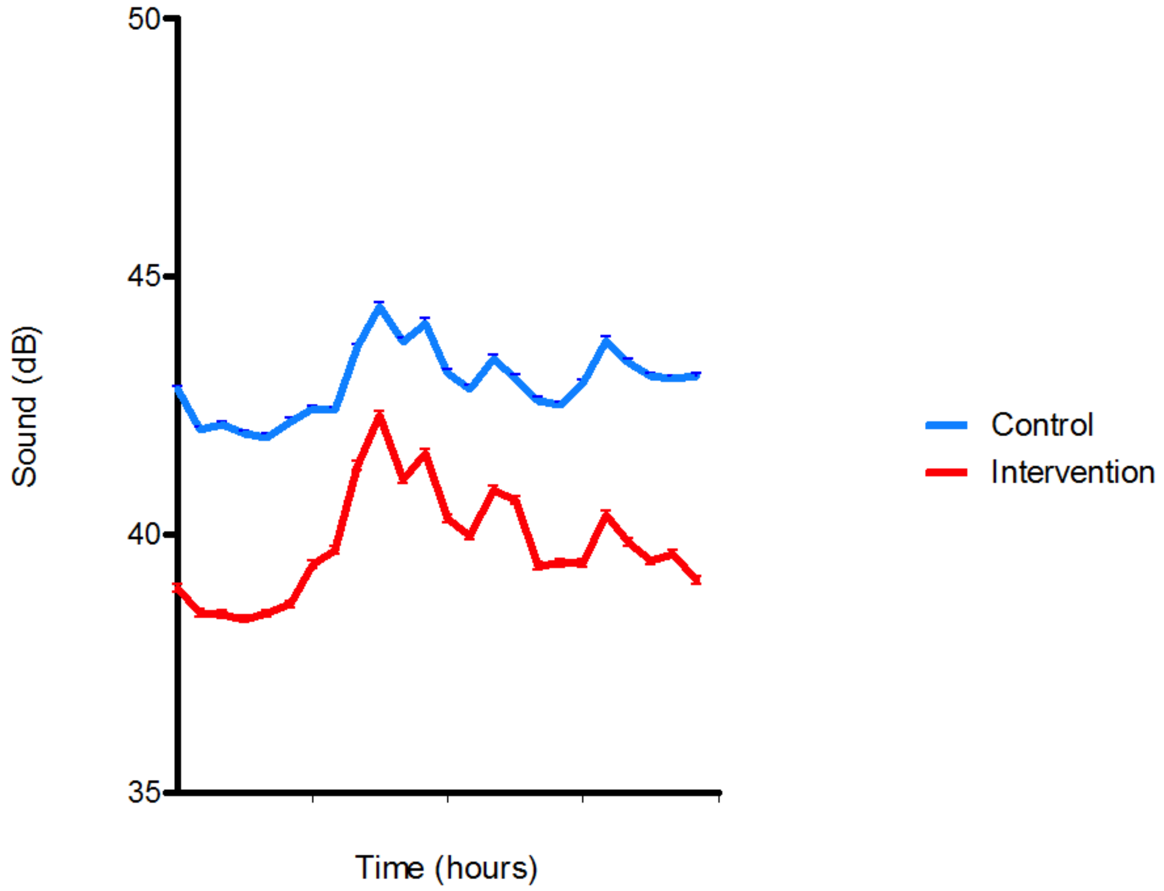

Daily mean dB levels (LaEQ-dB) depicted over 24 hours (2 devices, N= 2601 measurement days).
Data in the figure are expressed as mean and SEM.
